# B-cell intrinsic RANK signaling cooperates with TCL1 to induce lineage-dependent B-cell transformation

**DOI:** 10.1038/s41408-024-01123-6

**Published:** 2024-08-28

**Authors:** Lisa Pfeuffer, Viola Siegert, Julia Frede, Leonie Rieger, Riccardo Trozzo, Niklas de Andrade Krätzig, Sandra Ring, Shamim Sarhadi, Nicole Beck, Stefan Niedermeier, Mar Abril-Gil, Mohamed Elbahloul, Marianne Remke, Katja Steiger, Ruth Eichner, Julia Jellusova, Roland Rad, Florian Bassermann, Christof Winter, Jürgen Ruland, Maike Buchner

**Affiliations:** 1 Institute of Clinical Chemistry and Pathobiochemistry, TUM School of Medicine and Health, Technical University of Munich, Munich, Germany; 2 TranslaTUM - Central Institute for Translational Cancer Research, Technical University of Munich, Munich, Germany; 3 German Cancer Consortium (DKTK), partner site Munich, Munich, Germany; 4 Department of Medical Oncology, Jerome Lipper Multiple Myeloma Center, Dana Farber Cancer Institute, Boston, MA USA; 5 Harvard Medical School, Boston, MA USA; 6 Broad Institute of MIT and Harvard, Cambridge, MA USA; 7 Department of Medicine III, TUM School of Medicine and Health, Technical University Munich, Munich, Germany; 8 Institute of Molecular Oncology and Functional Genomics, TUM School of Medicine and Health, Technical University of Munich, 81675 Munich, Germany; 9 Institute of Pathology, Technical University Munich, Munich, Germany; 10 German Cancer Research Center (DKFZ), Heidelberg, Germany; 11 Bavarian Center for Cancer Research (BZKF), Munich, Germany; 12 German Center for Infection Research (DZIF), partner site Munich, 81675 Munich, Germany

**Keywords:** Cancer models, Lymphoproliferative disorders, Myeloma, Plasma cells

## Abstract

B-cell malignancies, such as chronic lymphocytic leukemia (CLL) and multiple myeloma (MM), remain incurable, with MM particularly prone to relapse. Our study introduces a novel mouse model with active RANK signaling and the TCL1 oncogene, displaying both CLL and MM phenotypes. In younger mice, TCL1 and RANK expression expands CLL-like B1-lymphocytes, while MM originates from B2-cells, becoming predominant in later stages and leading to severe disease progression and mortality. The induced MM mimics human disease, exhibiting features like clonal plasma cell expansion, paraproteinemia, anemia, and kidney and bone failure, as well as critical immunosurveillance strategies that promote a tumor-supportive microenvironment. This research elucidates the differential impacts of RANK activation in B1- and B2-cells and underscores the distinct roles of single versus combined oncogenes in B-cell malignancies. We also demonstrate that human MM cells express RANK and that inhibiting RANK signaling can reduce MM progression in a xenotransplantation model. Our study provides a rationale for further investigating the effects of RANK signaling in B-cell transformation and the shaping of a tumor-promoting microenvironment.

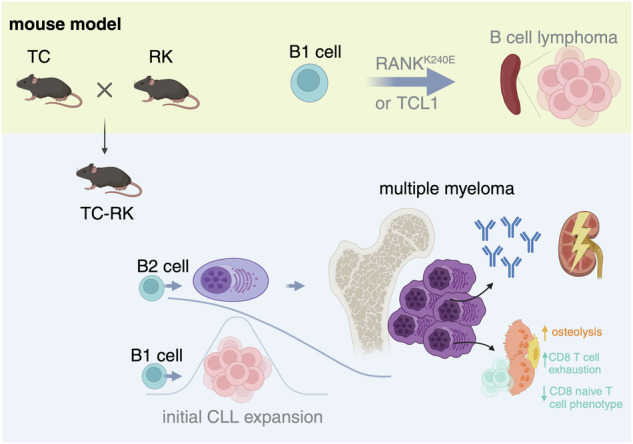

## Introduction

B lymphocytes are vital components of the adaptive immune system, orchestrating responses to pathogens through antibody production and memory cell formation [1]. However, their dysregulation can lead to various hematological malignancies, including B-cell lymphomas and multiple myeloma (MM), highlighting the delicate balance between immune competence and oncogenesis [2, 3].

A critical player in B-cell malignancies is the NF-κB signaling pathway, essential for cell survival, differentiation, proliferation, inflammation, and immune regulation. Aberrant NF-κB activation, commonly observed in these malignancies, can result from interactions with the tumor microenvironment or mutations in upstream factors or inhibitors [4]. Within the TNF receptor superfamily (TNFRSF), the receptor activator of NF-κB (RANK; *TNFRSF11A*) has emerged as a significant factor in B-cell immunopathology [5–7]. Understanding how RANK activation interacts with other oncogenic factors in a lineage-specific manner to promote B-cell transformation remains incomplete, underscoring a broader challenge in deciphering the pathogenesis of B-cell-derived malignancies across different B-cell lineages.

Among these malignancies, chronic lymphocytic leukemia (CLL) and MM are prevalent and have rising incidences in Western countries [8]. Recurrent mutations in the RANK encoding gene *TNFRSF11A*, specifically RANK^K240E^, found in diffuse large B-cell lymphoma [5], lead to significant expansion in the B1-cell subset, innate-like, self-renewing B lymphocytes in mice. Upon aging, the RANK-driven B1-cells transform into a CLL-like disease. Furthermore, blocking RANK in both human CLL and the TCL1-driven CLL mouse model reduces disease progression in preclinical models, confirming the relevance of RANK signaling for CLL progression [9]. While murine CLL-like disease origins from B1-cells, MM arises from B2-cells, specifically from the terminally differentiated plasma cells responsible for producing antibodies [10–12]. In myeloma, these cells undergo malignant transformation, leading to uncontrolled proliferation and accumulation within the bone marrow and secretion of abnormal monoclonal antibodies.

The microenvironment of the bone marrow has been shown to play an important role in promoting tumorigenesis in multiple myeloma. Interaction of myeloma cells with bystander cells activates pro-survival and pro-proliferation signaling [13]. Various environmental cues within the bone marrow niche have the capacity to activate canonical and non-canonical NF-κB signaling and promote tumor progression [14]. B-cell activating factor (BAFF), a cytokine that induces non-canonical NF-κB activation, is thought to support multiple myeloma cell survival and to contribute to poor disease progression [15]. RANK signaling is equally able to activate the non-canonical NF-κB pathway, but its role in MM development or propagation is poorly understood to date. Research on myeloma has primarily focused on RANK’s role in promoting bone disease, where RANK ligand (RANKL) is secreted by myeloma and stromal cells to promote osteoclastogenesis [16]. While clinical data on the RANKL-blocking antibody denosumab suggests a potential relevance of RANK signaling in disease progression [17], its direct involvement in myeloma pathogenesis remains to be explored.

Furthermore, while CLL therapies often induce long-term responses by targeting B-cell receptor (BCR) signaling pathways or BCL2 [18, 19], MM treatments frequently face early relapse despite strategies to eliminate malignant plasma cells and modulate the bone marrow microenvironment to restore normal hematopoiesis and immune function [20]. This highlights the need for a deeper understanding of the factors contributing to myeloma formation, drug resistance, and bone marrow niche homing to overcome the limitations of existing therapeutic strategies.

In our previous work we have shown that increased RANK signaling boosts differentiation towards the B1-cell stage which ultimately gives rise to CLL in aged animals [9]. Here we show that the simultaneous activation of RANK signaling and TCL1, a factor known to induce CLL, does not simply accelerate CLL development but enables the outgrowth of aggressive multiple myeloma. We find that mice with a simultaneous increase in RANK signaling and TCL1 expression initially develop B1-cell-derived CLL, which is however later suppressed by a more aggressive B2-cell-derived multiple myeloma outgrowth. The induced disease shows major features of human myeloma. Moreover, we demonstrate that human MM cells express RANK and that RANKL blockade can slow myeloma progression in a xenotransplantation mouse model. Combined, these findings demonstrate that depending on the signaling context RANK activation can give rise to malignancies originating from different B-cell subsets and provides a rationale to further explore possible tumor-promoting roles of RANKL expressing cells in the tumor-microenvironment of MM.

## Results

### Differential onset and progression of CLL and MM in a genetically induced mouse model

**Fig. 1:**
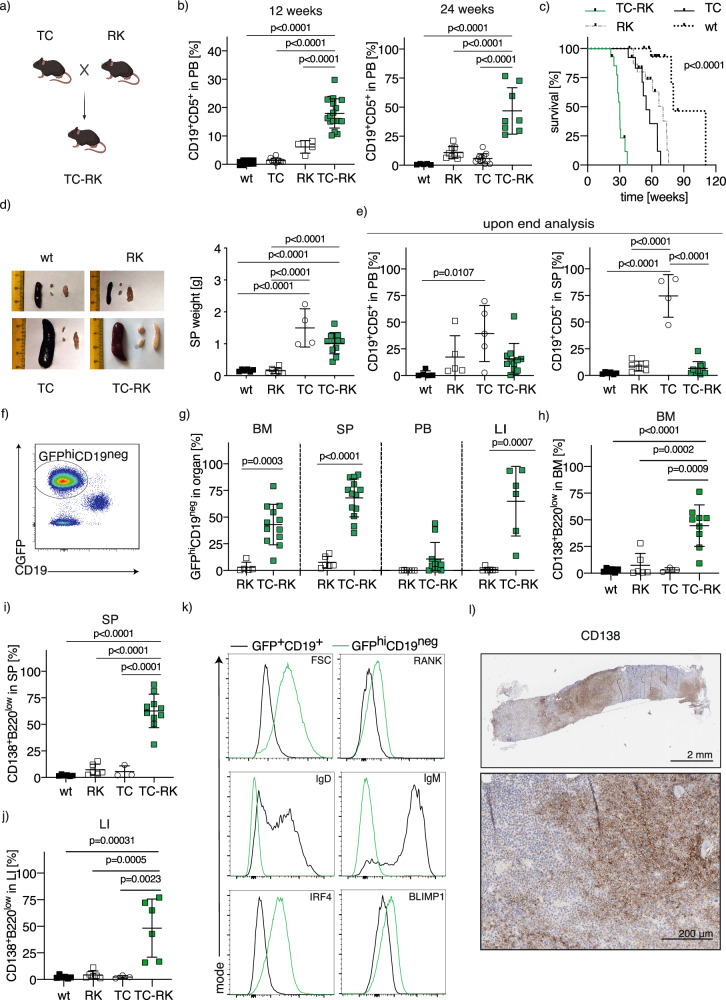
Active RANK and TCL1 signaling in B-cells causes a plasma cell disorder. **a** Breeding scheme to generate TC-RK mice. Scheme was generated with BioRender.com. **b** Percentage of CD19^+^CD5^+^ of total viable lymphocytes in the peripheral blood of 12- and 24- week-old animals (*n* = 3–17 per genotype) determined by flow cytometry. Wildtype and CD19^Cre^ animals are pooled as wt. Data was pooled from more than three experiments. **c** Kaplan-Meier overall survival analysis of TC-RK (*n* = 14), TC (*n* = 13), RK (*n* = 16) and wt (*n* = 17) control mice. For statistical analysis, *p* value of the log-rank test is shown. **d** Macroscopic appearance of representative spleens and lymph nodes (left) and dot plot graph (right) depicts spleen (SP) weight in gram (g) of diseased or aged mice with indicated genotypes (*n* = 4–11 per genotype). **e** Percentage of CD19^+^CD5^+^ of viable cells in the peripheral blood (PB, left, *n* = 5–11 per genotype) and SP (right, *n* = 5–13 per genotype) from animals with indicated genotypes upon signs of disease or aged mice. **f** Representative flow cytometric analysis of GFP and CD19 expression on viable splenocytes from a diseased TC-RK mouse. **g** Percentages of GFP^hi^CD19^neg^ cells of viable cells from BM, SP, PB and LI isolated from diseased TC-RK mice (*n* = 6–13 per organ) compared to aged RK mice (*n* = 5–6 per organ). **h** Percentages of CD138^+^B220^low^ cells of viable cells from bone marrow (BM) from diseased TC-RK mice and diseased or aged control mice (*n* = 3–10 per genotype). **i** Percentages of CD138^+^B220^low^ cells of viable splenocytes cells from diseased TC-RK mice and diseased or aged control mice (*n* = 3–9 per genotype). **j** Percentages of CD138^+^B220^low^ cells of viable cells from liver (LI) of diseased TC-RK mice and diseased or aged control mice (*n* = 3–6 genotype). **k** Representative histograms of forward scatter area (FSC-A), surface RANK, CD138, B220, IgM and IgD, as well as intracellular IRF4 and BLIMP1 from GFP^hi^CD19^neg^ cells (green) compared to GFP^+^CD19^+^ cells (black) from TC-RK mice. **l** Representative images of CD138 immunohistochemistry from bone of a diseased TC-RK mouse (scale bars: overview = 2 mm, detailed image with 20x magnification = 200 μm). Statistical analysis was performed using Student’s *t*-test and the one-way ANOVA with Tukey correction for multiple comparison. The *p* values are indicated in respective graphs. All data are presented as mean ± standard deviation.

To investigate the impact of active RANK signaling on the acceleration of TCL-1 induced lymphomagenesis in vivo, we generated triple-transgenic Eμ-TCL1 RANK^K240E^ CD19Cre mice (TC-RK) by crossing Eμ-TCL1 mice [21] (TC) with RANK^K240E^ CD19Cre [9] (RK) mice. These TC-RK mice express the diffuse large B-cell lymphoma (DLBCL)-derived hyperactive RANK^K240E^ human variant and enhanced green fluorescent protein (GFP) in B-cells, alongside TCL1, throughout B-cell development from immature to mature stages (Fig. 1a). Considering that both TC and RK mice models develop mature B-cell lymphoma originating from the B1-cell lineage, specifically chronic lymphocytic leukemia (CLL) [9, 21], we assessed B1-cell expansion in peripheral blood using CD19 and CD5 surface markers. As expected, we observed a significant increase in B1/CLL cells in TC-RK mice at 3 and 6 months, compared to mice expressing only a single transgene or the wild-type (including CD19^Cre/+^ littermate) controls (Fig. 1b).

TC-RK mice demonstrated a significantly decreased lifespan, marked by severe symptoms including accelerated breathing, diminished activity, and pronounced hepato- and splenomegaly, as shown by enlarged organ size and weight (Fig. 1c, d; Supplementary Fig. 1a, b). Unexpectedly, flow cytometric analysis of terminally ill TC-RK mice revealed low levels of CD19^+^CD5^+^ cells in both peripheral blood and spleen (Fig. 1e), suggesting that CLL cells might not directly contribute to the observed morbidity and mortality. Intriguingly, these mice showed a marked increase in GFP-expressing, CD19-negative (GFP^hi^CD19^neg^) cells in the bone marrow, spleen, and liver, indicating a deviation from the expected CLL phenotype (Fig. 1f–g). Further characterization of this population revealed high CD138 expression likely representing terminally differentiated plasma cells (Fig. 1h–j). This assertion is further corroborated by their increase in cell size, low levels of surface immunoglobulins, IgM and IgD, and the detection of intracellular expression of IRF4 and BLIMP1, critical transcription factors for plasma cell differentiation (Fig. 1k, Supplementary Fig. 1c) [22–25]. Immunohistochemical analysis demonstrated that the bone marrow from TC-RK mice is characterized by focal plasma cell regions that disrupt normal hematopoiesis, closely mirroring the infiltration patterns observed in human myeloma (Fig. 1l). However, while normal plasma cells are typically confined to the bone marrow, their malignant counterparts in TC-RK mice infiltrated the spleen and liver, in some cases, the peripheral blood, mimicking the pathology of plasma cell leukemia in humans, which is associated with a poor prognosis [26, 27]. These results suggest that the pathology in TC-RK mice might closely resemble aggressive MM, with a significant expansion of plasma cells across various organs.

### TC-RK-induced disease reflects clinical features of human multiple myeloma

**Fig. 2:**
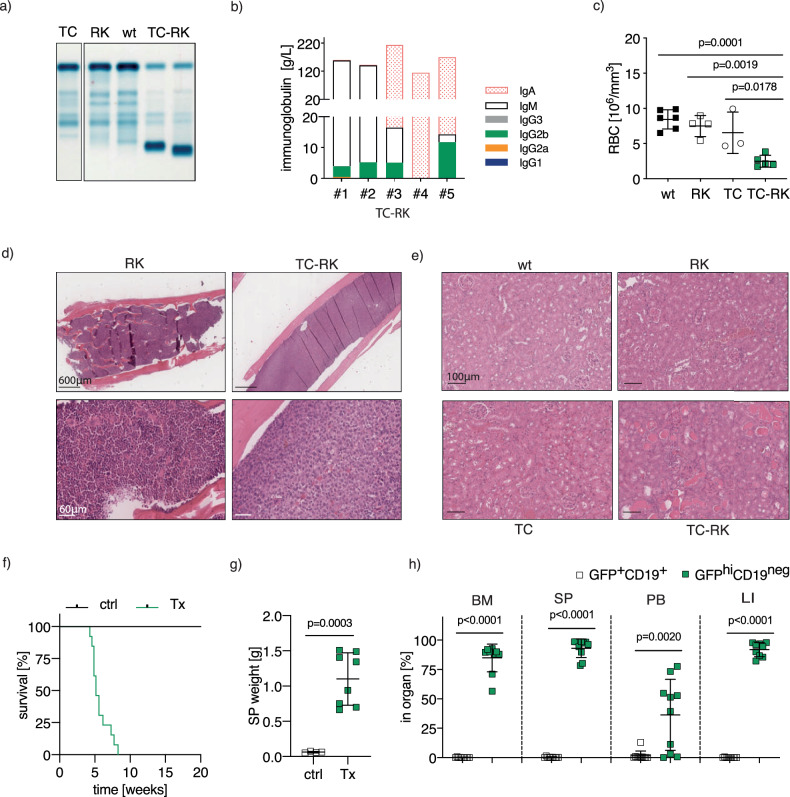
TC-RK induced disease mirrors features of human multiple myeloma. **a** Representative serum electrophoresis of immunoglobulins from TC-RK and control mice. **b** Representative quantification of immunoglobulin isotypes in plasma samples of five diseased TC-RK mice by flow cytometry-based multiplex immunoassay. **c** Red blood cell count (RBC) of diseased TC-RK mice and indicated controls (*n* = 3–6 per genotype). **d** Representative images of H&E staining from bone marrow of five-month-old RK and TC-RK mice (scale bars: overview = 600 μm, detailed images: 60 μm) **e** Representative images of H&E staining from kidneys of diseased TC-RK mice and indicated diseased or aged control mice (scale bars: 100 μm). **f** Kaplan-Meier OS analysis of Rag2^ko^ mice (*n* = 13) transplanted with TC-RK splenocytes derived from four different sick donor mice compared to Rag2^ko^ control mice. **g** Spleen weight of Rag2^ko^ mice (*n* = 8) transplanted with TC-RK splenocytes at end point and aged-matched Rag2^ko^ mice (*n* = 4). **h** Percentages of GFP^hi^CD19^neg^ cells and GFP^+^CD19^+^ cells of viable cells from BM, SP, PB and LI isolated from diseased Rag2^ko^ mice (*n* = 9–10 per organ) transplanted with TC-RK splenocytes derived from three different donor mice. Statistical analysis was performed using Student’s *t*-test and one-way ANOVA with Tukey correction for multiple comparison. *P* values are indicated in respective graphs and all data are presented as mean ± standard deviation.

To further investigate the potential characteristics of multiple myeloma in our mouse model, we performed serum protein electrophoresis to check for immunoglobulin secretion by plasma cells in TC-RK mice, a diagnostic and monitoring tool for plasma cell malignancies in clinical settings [28]. Remarkably, we observed distinct M-spikes exclusively in the serum of TC-RK mice, indicating the presence of monoclonal immunoglobulins (Fig. 2a). Further analysis of immunoglobulin isotypes revealed that the expanded plasma cell clones in TC-RK mice predominantly secreted IgM, IgG2b, or IgA (Fig. 2b), suggesting that both pre-germinal center and class-switched B-cells contribute to the clonally expanded plasma cell pool.

We also assessed for symptoms typical of human myeloma in TC-RK mice, such as anemia, bone lesions, and kidney failure. At the onset of disease symptoms, TC-RK mice exhibited significantly lower red blood cell counts compared to controls (Fig. 2c). Bone histology in ill TC-RK mice revealed myeloma-like changes, including osteolytic lesions adjacent to multifocal plasma cell infiltrations and altered spongiotic architecture in the myeloma-infiltrated regions, as evaluated by a certified pathologist, confirming the induction of a myeloma-like disease in these mice (Fig. 2d). Kidney analysis showed severe renal disease characterized by tubular degeneration, intratubular protein aggregates, and the presence of intravascular tumor cells (Fig. 2e). Conversely, control and TC mice displayed normal kidney histology, while RK mice showed signs of mild to moderate protein accumulation, possibly mediated by autoimmune immunoglobulin aggregates [9]. Spleen architecture was also disrupted by the severe expansion of CD138/IRF4^+^ plasma cells (Supplementary Fig. 1d). To also test the disease-inducing potential of TC-RK-derived plasma cells, we injected TC-RK-derived splenocytes into Rag2^KO^ mice, which are devoid of both B and T-cells, resulting in lethal disease in all recipients (Fig. 2f). These mice showed symptoms requiring euthanasia 5 to 10 weeks post transplantation and organ analysis revealed a significant increase in GFP^hi^CD19^neg^ plasma cells in the bone marrow, spleen, and liver (Fig. 2g, h). Plasma cells were detectable in the blood only in cases where the donor mouse exhibited a leukemic phenotype. Bone marrow derived plasma cells were equally efficient in promoting disease burden (Supplementary Fig. 1e, f).

These findings collectively demonstrate that TC-RK mice exhibit a clonal plasma cell expansion highly similar to human myeloma with full penetrance by 7-months of age. This malignancy impacts bone niches, leading to anemia, osteolytic changes, and significant renal damage. Importantly, this disease is transplantable from affected to unaffected mice, underscoring its value as a model for studying the characteristics of multiple myeloma.

### B2 B-cells are the cell-of-origin of TC-RK driven multiple myeloma

**Fig. 3:**
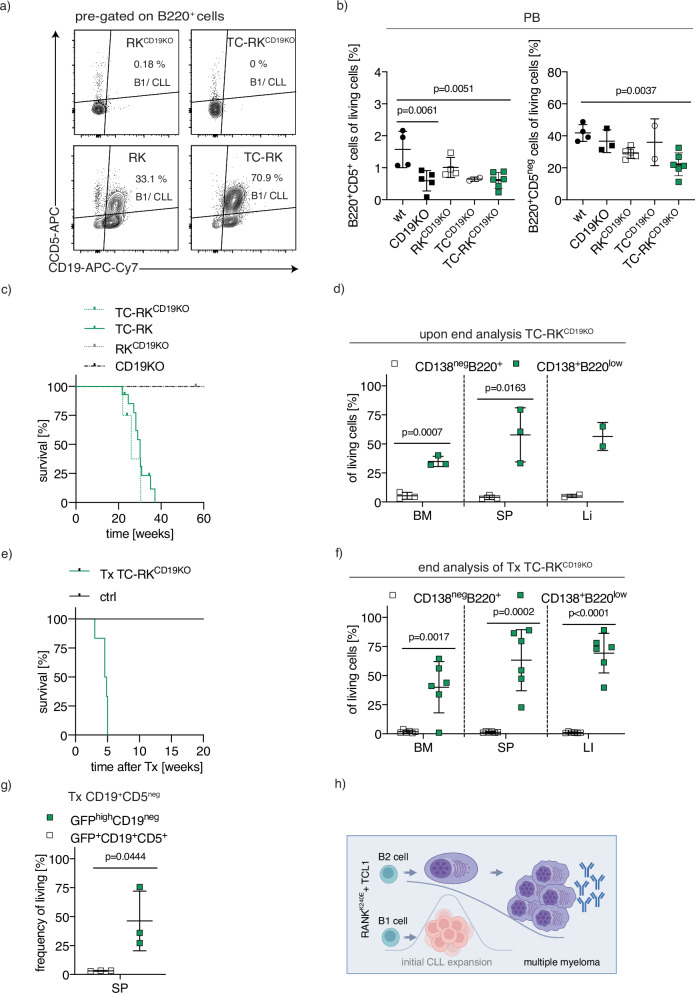
Origin of myeloma cells in TC-RK driven mouse models. **a** Representative flow cytometric analysis of CD5 and CD19 expression on B220^+^ splenocytes from mice with indicated genotypes. **b** Percentage of B220^+^CD5^+^ and B220^+^CD5^neg^ cells of total viable lymphocytes in the peripheral blood from animals with indicated genotypes (*n* = 2–6 per genotype) determined by flow cytometry. Pooled data from three different experiments. **c** Kaplan-Meier OS analysis of TC-RK^CD19KO^ (*n* = 4) mice compared to TC-RK (*n* = 13) and RK^CD19KO^ (*n* = 3) and CD19KO (*n* = 5) mice. **d** Percentages of CD138^+^B220^low^ and B220^+^CD138^neg^ cells of viable cells from BM, SP and LI isolated from diseased TC-RK^CD19KO^ mice (*n* = 3). **e** Kaplan-Meier OS analysis of Rag2^ko^ mice (*n* = 6) transplanted with TC-RK^CD19KO^ splenocytes derived from two different sick donor mice compared to Rag2^ko^ control mice. **f** Percentages of CD138^+^B220^neg^ cells and B220^+^CD138^neg^ cells of viable cells from BM, SP and LI isolated from diseased Rag2^ko^ mice (*n* = 6) transplanted with TC-RK^CD19KO^ splenocytes derived from two different donor mice. **g** Percentages of GFP^hi^CD19^neg^ and GFP^+^CD19^+^ cells of viable splenocytes isolated from Rag2^ko^ mice (*n* = 3) after transplantation of sorted CD19^+^CD5^neg^ BM cells from three different 3-month-old TC-RK donor mice. **h** Graphical summary of B2-cells giving rise to MM cells and B1-cells giving rise to CLL cells in TC-RK mice. Graphic was generated with BioRender.com. Statistical analysis was performed using Student’s *t*-test and one-way ANOVA with Tukey correction for multiple comparison. *P* values are indicated in respective graphs and all data are presented as mean ± standard deviation.

Observing CLL-like B1-lymphocyte expansion in younger TC-RK mice prior to the overt onset of myeloma, we next aimed to explore the relationship between CLL and plasma cell populations. Given that myeloma typically originates from CD5-negative, post-germinal center B2-cells, whereas murine CLL is believed to arise from the B1-lineage that express CD5 [12], our goal was to examine the impact of the B1-cell compartment on myeloma development. We investigated this by deleting CD19 through homozygous CD19^Cre/Cre^ expression (CD19^KO^ mice) [29], which substantially reduces the B1-cell population [30]. We first verified that CD19 deletion halted the development of both CLL and B1-cells in TC-RK^CD19KO^ mice in the spleen and the blood, while the B2-cell development was less affected, still comprising approximately 20% of peripheral blood cells (Fig. 3a, b). Despite the lack of B1/CLL cell expansion in CD19^KO^ mice, expression of TC-RK still resulted in significant plasma cell expansion and transformation. These effects were comparable to those observed in heterozygous CD19-expressing TC-RK mice (Fig. 3c, d). The malignant phenotype of the plasma cells derived from CD19^KO^ TC-RK mice was further validated by their successful transplantation into Rag2^KO^ mice, leading to rapid disease onset (Fig. 3e, f). This highlights that TC-RK specifically facilitates myeloma development within the B2-cell lineage, irrespective of CLL formation and the B1-lineage. This finding was further corroborated by transplanting sorted B2 (CD19^+^CD5^neg^) bone marrow cells from young TC-RK mice into Rag2^KO^ mice. Again, these TC-RK-derived B2-cells progressed into myeloma in the absence of B1-cells and CLL (Fig. 3g), confirming the myeloma development is driven in B2-cells by TC-RK, independent of B1-cell involvement.

Therefore, our analysis reveals that TC-RK uniquely fosters myeloma originating from B2-cells, separate from the CLL phenotype (Fig. 3h). This underscore specific B2-cell-intrinsic mechanisms as pivotal for myeloma genesis and this delineation between the pathways leading to CLL and myeloma highlights the complexity of oncogenic events driving B-cell malignancies.

### Mechanistic insights into the role of RANK and TCL1 in driving myeloma formation

**Fig. 4:**
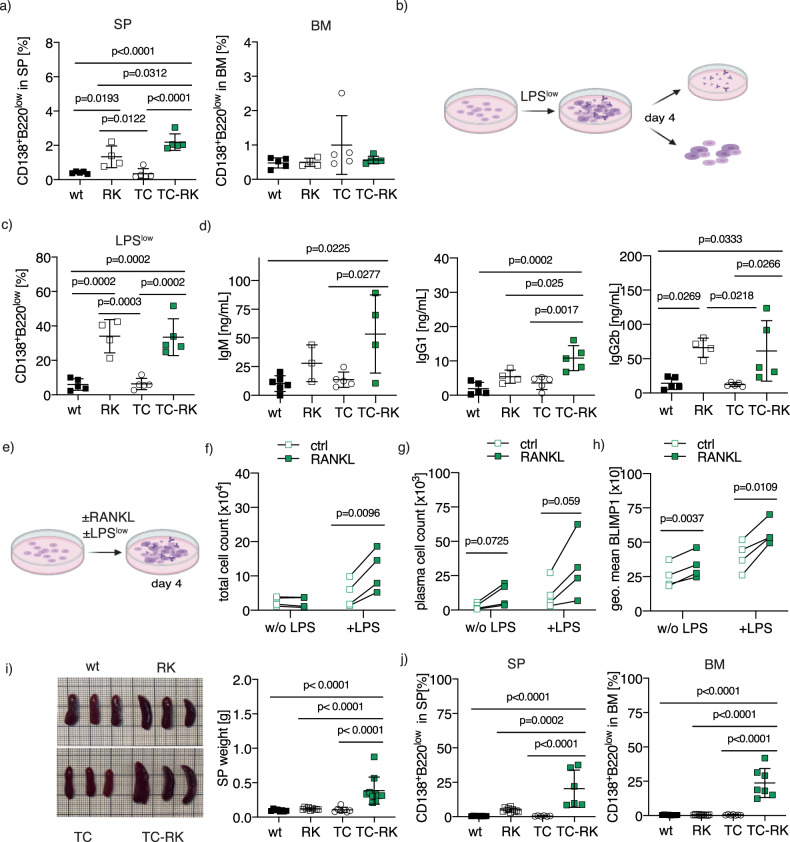
Active RANK signaling drives a plasma cell differentiation program via Blimp1 expression. **a** Percentages of CD138^+^B220^low^ plasma cells of viable cells from SP (left) and BM (right) of six-week-old TC-RK (*n* = 5) and control mice (*n* = 4–5 per genotype). Pooled data from three different experiments. **b** Experimental set up of in vitro differentiation of naive B-cells using low concentrations of LPS (LPS^low^, 100 ng/mL). B-cell stage and immunoglobulins in supernatants were analyzed by flow cytometry four days after stimulation. Graphic was created using BioRender.com. **c** Percentages of CD138^+^B220^low^ plasma cells after in vitro differentiation of naive B-cells derived from animals with indicated genotype (*n* = 4–5 per genotype) with LPS^low^ were determined by flow cytometry after four days. Biological replicates were pooled from two individual experiments. **d** Quantification of immunoglobulin isotypes in supernatants from in vitro differentiated naive B-cells, derived from animals with indicated genotype (*n* = 3–5 per genotype) and stimulated for four days with LPS^low^, by flow cytometry-based multiplex immunoassay. Pooled data from two independent experiments. **e** Experimental set up of in vitro differentiation of naive B-cells derived from TC-RK mice using recombinant RANKL in presence or absence of LPS^low^ for four days. **f** Total TC-RK-derived B-cell count (*n* = 4) per well after in vitro stimulation with RANKL and LPS^low^ as indicated. Biological replicates were pooled from two individual experiments. **g** TC-RK-derived CD138^+^B220^low^ plasma cell count (*n* = 4) per well after in vitro differentiation using RANKL and LPS^low^ as indicated. Biological replicates were pooled from two individual experiments. **h** Geometric mean (geo. mean) of BLIMP1 from living cells after in vitro differentiation of TC-RK-derived B-cells (*n* = 4) using RANKL and LPS^low^ as indicated. Biological replicates were pooled from two individual experiments. **i** Macroscopic appearance of representative spleens (left) and dot plot graph (right) depicts SP weight in gram (g) of 20-week-old TC-RK (*n* = 10) and aged-matched control mice (*n* = 7–9 per genotype). **j** Percentages of CD138^+^B220^low^ plasma cells of viable cells from SP (left) and BM (right) from 20-week-old TC-RK (*n* = 6–7) and aged-matched control mice (*n* = 6–10 per genotype). Pooled data from more than three experiments. Statistical analysis was performed using Student’s *t*-test and one-way ANOVA with Tukey correction for multiple comparison. *P* values are indicated in respective graphs. All data are presented as mean ± standard deviation.

To further elucidate myeloma development in the TC-RK mouse model, we examined the effects of transgene expression on B-cell differentiation and maturation in young mice. At six weeks, RANK and TCL1 transgenes did not significantly impact B-cell precursor development in the bone marrow (Supplementary Fig. 2a). However, significant increases in marginal zone B-cells and B1-cells were observed in the spleens of RK and TC-RK mice compared to TC and wt controls (Supplementary Fig. 2b, c). Notably, RK and TC-RK mice also showed a higher proportion of plasma cells in the spleen, though not in the bone marrow, than their TC and wt counterparts (Fig. 4a), suggesting that RANK transgene expression alone might promote splenic plasma cell differentiation. We therefore sought to assess the intrinsic potential for plasma cell differentiation of B-cells in vitro. Naive splenic B-cells (excluding B1-cells) from TC-RK and control mice were stimulated ex vivo (Fig. 4b). At low LPS concentrations in the absence of IL4, insufficient to induce plasma cell differentiation in wt and TC mice [31], B-cells from RK and TC-RK mice successfully differentiated into plasma cells and secreted higher levels of IgM, IgG2b, IgG1, and IgA (Fig. 4c, d; Supplementary Fig. 2d). This suggests that RK expression may mediate a non-canonical pathway to plasma cell differentiation and isotype switching, reducing the requirement for IL-4 signaling. The differentiation potential in RK-expressing B-cells was less pronounced following optimal combined LPS and IL-4 stimulation (Supplementary Fig. 2e, f). We also tested whether suboptimal levels of T-cell-dependent stimuli for plasma cell differentiation could cooperate with active RANK signaling, hypothesizing that active RANK signaling reduces the requirement for BCR/CD40 co-stimulation to initiate a comprehensive B-cell activation program for plasma cell generation. However, neither suboptimal BCR stimulation nor anti-CD40 stimulation alone induced meaningful plasma cell differentiation in RK-expressing B-cells (Supplementary Fig. 2g, h). This suggests that low levels of LPS, but not BCR or CD40 signaling, are sufficient to cooperate with RK signaling to trigger plasma cell differentiation in TC-RK mice.

As TLR-mediated signaling seems critical to mediate the full differentiation to plasma cells in RK-expressing B-cells, we next analyzed the effects of recombinant murine RANKL on the viability of LPS-stimulated, naive TC-RK-derived B-cells and found a significant boost in B-cell population viability, with a notable, though not statistically significant, increase in plasma cell numbers (Fig. 4e–g). The presence of RANKL also elevated BLIMP1 protein levels [32], crucial for terminal B-cell differentiation, irrespective of LPS presence (Fig. 4h), emphasizing the essential role of RANK in promoting B-cell viability and differentiation into the plasma cell lineage, thus facilitating cellular transformation in aged mice. To gain further insight into signals regulated by RANK activation and how RANK signaling converges with BCR stimulation, we used RANK-overexpressing murine BAL17 lymphoma B-cell lines to dissect pathway activation following acute RANK and BCR signaling. We found that RANKL stimulation activated the JNK and NF-κB pathways in RANK-overexpressing BAL17 cell lines, while BCR activation led to rapid SRC/PLCγ2/AKT/ERK pathway activation. Simultaneous activation of RANK and BCR signaling resulted in the activation of both pathways but did not show significant enhancement in our model (Supplementary Fig. 3a, b). This suggests that dual RANK/BCR stimulation cooperates but do not potentiate differential signaling events early after activation, eventually driving plasma cell differentiation in our model.

To further track plasma cell expansion in vivo, we analyzed five-month-old mice. At this age, TC-RK mice displayed significantly enlarged spleens and increased plasma cell populations in both the spleen and bone marrow, the latter being the primary expansion site for malignant myeloma cells (Fig. 4i, j). Together with our in vitro analysis, this suggests that active RANK signaling is vital for initiating plasma cell differentiation, while the concurrent expression of TCL1 markedly intensifies this effect in vivo, leading to rapid and extensive plasma cell expansion within the bone marrow niche. This prompted us to analyze specific alterations within the bone marrow that contribute to this progression, thereby deepening our understanding of the mechanisms driving myeloma pathogenesis.

### Transcriptional alterations within the bone marrow niche upon TC-RK-mediated myeloma progression

**Fig. 5:**
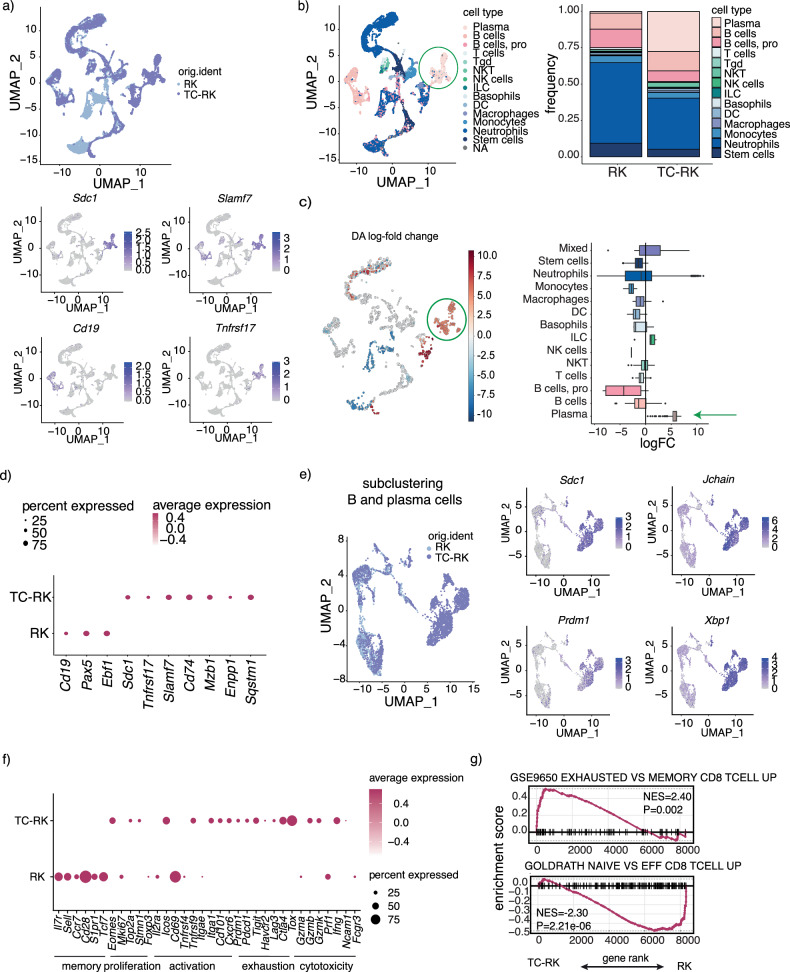
Landscape of the bone marrow microenvironment in TC-RK mice. **a** Uniform manifold approximation and projection (UMAP) visualizations of unsupervised clustering analysis of all cells that passed quality filtering. Cells are colored according to their genotype in the upper row and according to their *Sdc1*, *Cd19*, *Tnfrsf17* and *Slamf7* expression respectively below. **b** UMAP visualization of annotated clusters (left) and frequency of clusters (right) in RK (pooled from 3 mice) and TC-RK (pooled from 4 mice) derived BM cells. Plasma cells are highlighted with a green circle in the UMAP. **c** Differential abundance (DA) analysis for changes in cluster abundance (left) and log-fold change for each annotated cluster (right). Plasma cell cluster is highlighted by a green circle (left) or a green arrow (right). **d** Gene expression dot plot depicts the percent and average expression of B-cell markers within the B and plasma cell subcluster. **e** UMAP visualization of unsupervised clustering analysis of B-cells and plasma cells (defined by *Sdc1* and *Slamf7* expression). Cells are color coded according to their genotype (left) or according to *Sdc1*, *Jchain*, *Prdm1* and *Xpb1* expression respectively (right). **f** Gene expression profile visualize the percent and average expression of T-cell markers within the T-cell cluster. **g** Gene set enrichment analysis (GSEA) plot for CD8 T-cell exhaustion enriched T-cells from TC-RK mice (top) and GSEA plot of naive CD8 T-cells enriched in T-cells of RK mice (bottom).

To elucidate the changes within the bone marrow niche that facilitate myeloma cell proliferation and potentially evade immune responses, we performed single-cell RNA sequencing on bone marrow-derived cells from five-month-old TC-RK and RK mice. Our analysis revealed a pronounced shift in cell population dynamics in the bone marrow of TC-RK mice, with a marked increase in plasma cells identified by *Sdc1* (CD138), *Slamf7*, and *Tnfrsf17* (BCMA) expression and loss of *Cd19*, at expense of nearly all other immune cell types (Fig. 5a–c). Further detailed analysis within the B-cell/plasma cell population showed a diminished presence of B-cells marked by *Cd19* and *Pax5* expression in TC-RK mice, in stark contrast to the prevalent CD138/*Sdc1*^+^ plasma cells, also characterized by the expression of genes associated with myeloma, such as *Slamf7*, *Tnfrsf17* (BCMA)—a target for CAR-T-cell therapy—along with *Cd74*, *Mzb1*, *Enpp1*, and the autophagy gene *Sqstm1* (p62) (Fig. 5d). Furthermore, we detected high expression of the BLIMP1-encoding *Prdm1* gene and other genes associated with plasma cells, such as *Jchain* and *Xbp1* within the expanded plasma cell cluster (Fig. 5e). Additional B-cell (*Pax5*, *Ebf1*) and plasma cell markers (*Mzb1, Ptprc*^low^*)* as well as plasma cell scores are depicted in Supplementary Fig. 4a, b. Examination of the T-cell compartment exposed an increase in markers linked to T-cell activation/exhaustion (*Icos, Tnfrsf9, Pdcd1, Tigit, Tox*) and cytotoxicity (*Ifng, Gzmb, Gzmk*; Fig. 5f), with gene set enrichment analysis reinforcing the presence of an exhausted, effector phenotype among cytotoxic T-cells (Fig. 5g; scores depicted in Supplementary Fig. 4c). Therefore, our single-cell transcriptional profiling not only highlights a substantial expansion of the plasma cell lineage, enriched with myeloma-associated gene expressions within the bone marrow niche, but also underscores the presence of a significant activated and exhausted T-cell phenotype, reflecting potential roles in myeloma progression.

### Comparative analysis of TC-RK induced with myeloma of other murine and human origin

**Fig. 6:**
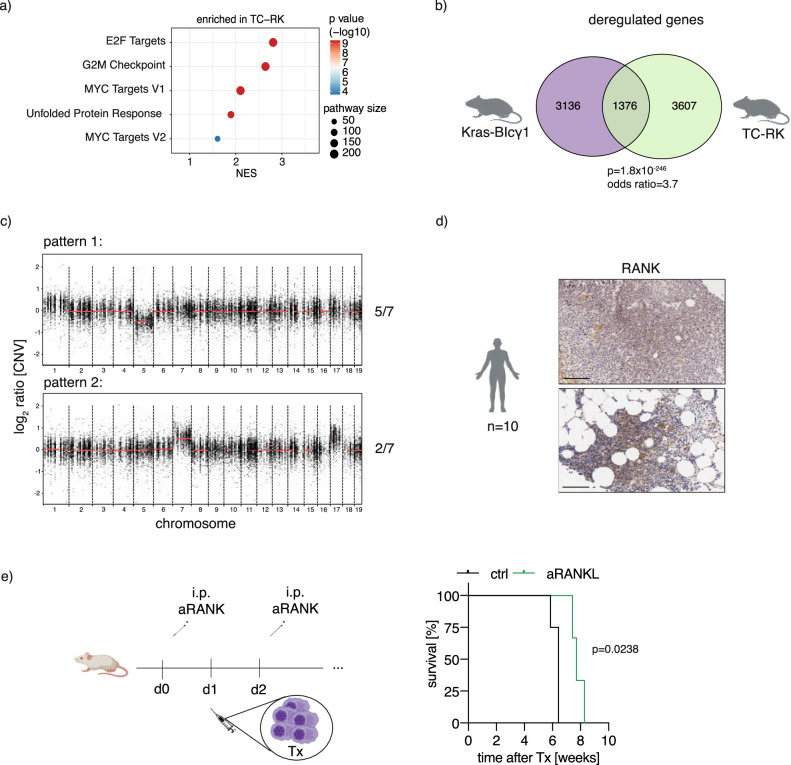
Comparative analysis between TC-RK induced myeloma and both other murine MM mouse models and human disease. **a** GSEA analysis showing hallmarks enriched in splenic plasma cells of TC-RK mice compared to plasma cells of RK mice. NES, normalized enrichment score. **b** Venn diagram depicts the overlap of deregulated genes in myeloma cells from Kras-BIγ1 mice [33] and TC-RK mice. Fisher’s exact test was applied and both *p* value and odd ratio are depicted. Illustrations were generated using BioRender.com. **c** Representative copy number variation patterns of myeloma cells from TC-RK mice (*n* = 7). **d** Representative immunohistochemistry staining of primary patient biopsies confirmed RANK expression in myeloma cells. Illustrations were generated using BioRender.com. **e** Treatment schedule of xenograft model with anti-RANKL antibody prior and after injection of L363 in NSG mice (top) and Kaplan-Meier survival analysis (bottom) is shown for NSG mice received anti-RANKL (*n* = 3) or vehicle (*n* = 4) therapy prior and after L363 transplantation (Tx L363). Statistical significance with corresponding *p* values calculated by log-rank (Mantel-Cox) test is depicted in the graph. Graph represents one out of three independent experiments.

To determine whether our new myeloma model shares additional features to previously described models, we next compared the transcriptional profile of (pre-)malignant plasma cells of TC-RK mice with those of RK mice at five months age using bulk RNA sequencing analysis and subsequently performed gene set enrichment analysis (Fig. 6a). This revealed upregulation of several pathways, most of which were previously identified in both newly developed murine myeloma models and in human myeloma [33]. These include upregulation of E2F transcription factor-related gene expression and a MYC signature, pathways that have recently been identified as responsive to targeted therapies in myeloma [33]. Notably, we also observed an enhanced unfolded protein response (UPR) pathway, presumably triggered by the substantial immunoglobulin production in TC-RK plasma cells. A detailed cross-comparison with other myeloma models [33] showed pronounced parallels (Fig. 6b, Supplementary Fig. 4a), most notably with the model induced by KRAS^G12D^ in conjunction with BCL2 overexpression, which exhibited the highest similarity (*p* = 1.8 × 10^−246^; odds ratio (OR) = 3.7). Furthermore, when comparing the transcriptomic profiles of TC-RK induced myeloma with human myeloma cases, there was a notable correspondence, particularly in the upregulation of myeloma-associated genes such as *Birc5*, *Ezh2*, *Prc1*, and *Foxm1* (*p* = 1.2 × 10^−5^; OR = 1.7), and in the downregulation of genes like *Cd19*, *Ctsh*, and *Tmsb4x* (*p* = 1.7 × 10^−4^; OR = 1.6; Supplementary Table 1) [34, 35].

Using whole exome sequencing, we discovered that a substantial proportion of the mutated genes in TC-RK myeloma cells, specifically 44% (72 out of 164), have previously been identified in human myeloma datasets [36] (*p* = 1.7 × 10^−6^; OR = 2.1), underscoring a notable genetic similarity between our model and human myeloma. Additionally, 23% of the mutations in our study (*p* = 3.1 × 10^−10^; OR = 3.8) were found in genes that are recurrently mutated in human myeloma (defined as a mutation present in at least 2 patients [36]) including the BCL6 interacting factor Dnah9 [37, 38] and the immunomodulating factor Irf2bp2 (Supplementary Table 2) [39, 40]. These findings suggest a significant overlap in the mutational landscape between our model and the genetic alterations observed in human myeloma, reinforcing the relevance of our model in the context of human disease. We also explored copy number variations (CNV) in our dataset, revealing two mutually exclusive CNV patterns previously identified in murine myeloma models [33, 41]. Specifically, we observed recurrent gains in chromosomes 7 and 17 in five out of seven TC-RK-derived myelomas [33]. The remaining two mice demonstrated a loss of chromosome 5 (Fig. 6c), consistent with other reports in the myeloma literature [33, 41]. While the precise impact of these chromosomal changes warrants further investigation, their presence in our model and others confirms overlapping disease evolutions.

Given the parallels between our TC-RK model and human myeloma, we explored the potential role of RANK signaling in human myeloma. Analysis of bone marrow immunohistochemistry from myeloma patients revealed stable RANK expression in most cases (Fig. 6d; Quantification in Supplementary Fig. 4b). We tested the functional significance of RANK signaling by treating L363-xenotransplanted mice, finding that early RANKL blockade significantly slowed myeloma progression and extended disease-free survival in this aggressive model (Fig. 6e). These findings underscore a potential relevance of B-cell intrinsic RANK in human myeloma and clarify the previously suggested anti-myeloma effects of denosumab, which targets RANKL in the treatment of bone disease [42].

Our findings collectively demonstrate that active RANK signalling in combination with TCL1 initially promote CLL expansion which is predominated by myeloma formation that accurately mirrors key aspects of the human disease. Characterized by a rapid onset of myeloma within seven months and complete penetrance, this model stands as a significant advancement in simulating critical disease features. Its efficiency and reliability offer an invaluable tool for further exploring immunosurveillance processes and possible resistance mechanisms in myeloma, underscoring its potential to significantly impact future research and therapeutic strategies.

## Discussion

In this study, we introduce a novel mouse model that reveals an unexpected phenotype of initial CLL expansion followed by aggressive myeloma formation through constitutive RANK signaling and TCL1 overexpression. This model accurately reflects the phenotypic, transcriptional, and genomic hallmarks of other murine myeloma models [33], while overcoming limitations of previous models that exhibited delayed onset and inconsistent disease development [43–45]. Our model not only provides insights into the complex consequences of lineage-specific oncogene expression but also serves as a valuable tool for precise monitoring of immunosurveillance dynamics and evaluating potential therapeutic interventions. Furthermore, our data suggest a significant contribution of RANK signaling to the pathology of human multiple myeloma, underscoring the potential for targeted therapies in this context.

We demonstrate that oncogenes driving B-cell lymphoma, specifically TCL1 and the mutant RANK^K240E^, synergize to initially drive CLL progression, yet invariably shift towards a myeloma phenotype originating from the B2-cell lineage. The interplay between specific oncogenes and the cellular context in which they are expressed plays a critical role in the pathogenesis of malignancies. Our findings underscore that the manifestation of disease can vary significantly depending on the cell type harbouring the oncogene and the presence of additional oncogenic events. For instance, in our study, the expression of TCL1 and active RANK in B2-cells specifically led to the development of multiple myeloma, independent of the CLL-like phenotype observed with similar oncogene expression in B1-cells.

Mechanistically, we discovered that low LPS levels are sufficient to induce plasma cell differentiation in RK- and TC-RK-derived B2-cells, but not wild type B-cells. In contrast, BCR signaling alone was insufficient to drive RK-expressing B-cells into the plasma cell lineage.

LPS activates B-cells via TLR4 in a polyclonal manner and induces plasma cell differentiation in vitro. In contrast, anti-BCR stimulation in vitro leads to B-cell activation and proliferation but does not promote differentiation of wild-type B-cells into plasma cells. We chose these stimuli to test whether RK- or TC-RK expression amplifies plasma cell differentiation signals or replaces them. Since we did not observe plasma cell differentiation upon BCR activation in TC-RK expressing B-cells, we conclude that TC-RK expression lowers the threshold for signals needed to commit to the plasma cell fate but is not sufficient to replace these signals. Additionally, we stimulated CD40 and assessed plasma cell differentiation. In vivo, B-cells are exposed to CD40L during immune responses to thymus-dependent antigens, inducing pro-survival signaling. In vitro, CD40 stimulation alone induces low levels of plasma cell differentiation, but the signal is substantially weaker than LPS stimulation. For comparison, the frequency of plasma cells in wild-type B-cells stimulated with suboptimal LPS levels was around 5%, but only 1% with CD40 stimulation. TC-RK expressing B-cells showed a trend towards increased plasma cell differentiation compared to B-cells expressing TC alone; however, overall plasma cell differentiation remained low. This suggests that while TC-RK signaling can enhance plasma cell differentiation upon various stimulations, the strength of the differentiation signal is crucial for the final outcome.

Consistent with our functional assays, we found that RANK signaling does not alter the quality of BCR signaling but provides the cells with additional pro-survival signals. To study RANK induced signaling we have transduced BAL17 B lymphoma cells with the wildtype and constitutively active form of RANK (RANK^wt^ and RANK^K240E^, respectively). We chose BAL17 cells since they do not express RANK and BAL17 cells transduced with an empty vector can thus serve as a negative control for RANK-mediated signaling. Treatment with RANKL induced phosphorylation of the NF-κB pathway signaling molecule IKKα/β as well as JNK phosphorylation in both RANK^wt^- or RANK^K240E^- expressing cells but not BAL17 cells transduced with an empty vector. Activation of NF-κB is critical for driving human myeloma progression [14]. In mouse models, active IKKβ in combination with pro-survival signals has been found to drive multiple myeloma [33], highlighting the importance of NF-κB activation in driving the disease. Active JNK phosphorylates nuclear transcription factors such as c-Jun and ATF2, leading to the transcription of target genes that protect myeloma cells from apoptosis [46]. Additionally, JNK activation by BLyS, another TNFR superfamily member, creates a positive feedback loop that enhances cell survival and proliferation in myeloma-derived plasma cells [47]. In summary RANK signaling increases critical downstream effectors with known relevance in driving myeloma formation.

Importantly we found, the phosphorylation of IKK and JNK to be higher in cells expressing RANK^K240E^ rather than RANK^wt^. This suggests that while wildtype RANK can provide B-cells with pro-survival signaling, the hyperactive form is more efficient in doing so. Consistent with our signaling studies, our functional experiments indicate that wild-type RANK provides multiple myeloma cells with survival signals, as blocking RANK signaling in multiple myeloma xenotransplantation models reduces tumor burden. To test whether RANK activation modulates BCR dependent signaling, we stimulated the cells with an anti-BCR antibody alone or in combination with RANKL treatment. Early events of BCR activation such as SRC/PLCγ/AKT and ERK phosphorylation were not altered by RANK^wt^- or RANK^K240E^ expression, suggesting no direct regulation of BCR signaling via RANK.

Therefore, our study demonstrates that RANK hyperactivation increases pro-survival signaling via NF-κB and JNK and amplifies signals inducing plasma cell differentiation. Previous studies have shown that RANK is not required for the activation, proliferation, and differentiation of normal B-cells [48]. Our findings reveal that RANK hyperactivation boosts plasma cell differentiation in vitro. This suggests that while RANK is not essential, it can enhance plasma cell differentiation. In the context of malignant transformation, RANK hyperactivation alone can drive CLL development in a mouse model [9]. However, multiple myeloma only develops in combination with additional pro-survival signals, such as those provided by TCL1. Our signaling studies indicate that RK and TC expression alone is insufficient to induce plasma cell development without differentiation signals. Thus, we hypothesize that RANK lowers the threshold for both survival and plasma cell differentiation. B1-cells are known to exhibit increased survival properties compared to B2-cells, making RANK expression alone sufficient to drive their malignant transformation into CLL. In contrast, transforming B2-cells may require additional survival signals, provided by TCL1 in our model. A key question remains why TC-RK induces multiple myeloma rather than DLBCL, where RANK^K240E^ expression was discovered [5]. Our studies demonstrate that Blimp1 expression and plasma cell differentiation are enhanced by RANK^K240E^ upon plasma cell differentiation stimuli. Future research should investigate whether low levels of TLR ligand exposure, e.g., from gut microbiota, initiate multiple myeloma development in this mouse model. Additionally, it would be interesting to assess whether inhibiting plasma cell differentiation by deleting Blimp1 in our mouse model would result in DLBCL rather than multiple myeloma development.

Interestingly, the transition to the severe myeloma phenotype is marked by a striking absence of CLL cells, suggesting competition for growth and/or survival limiting factors such as nutrients [49] or cytokines like BAFF, potentially facilitated through BCMA expression and its shedding [50], a hypothesis that warrants further exploration. This observation suggests a complex interplay of cellular and molecular factors influencing disease progression and highlights not only the cell-specific actions of oncogenes but also suggests that the co-occurrence of other molecular abnormalities may amplify or modify the oncogenic potential of a given gene. This complexity is crucial for understanding the full spectrum of oncogene-driven pathologies and indicates that targeted therapies must consider not just the oncogenic drivers but also the cellular and molecular context of their expression.

Emerging evidence, from our study and beyond, reinforces the potential of the RANKL-blocking antibody denosumab, not only for its bone-protective properties in late-stage myeloma, but also for its potential anti-myeloma effects. Recent clinical studies demonstrate that denosumab therapy is associated with significantly better progression-free survival rates over those receiving zoledronic acid, which is similar effective in preventing bone loss [17]. Our findings, alongside other preclinical research [51–53], highlight the importance of B-cell intrinsic RANK signaling in the progression of myeloma and support denosumab’s capability to interfere in these processes. This insight is particularly relevant for the ongoing denosumab clinical trial in smoldering myeloma (Eudra-CT 2018-000924-32) and future applications of combination treatments. Notably, our model also highlights high expression of the scaffolding protein SQSTM1/p62, a factor promoting RANK signalling [54] but also conferring resistance to proteasome inhibitors in myeloma [55, 56]. Further insight into the regulation of p62 and RANK signaling may unveil novel strategies to counteract drug resistance in myeloma.

In conclusion, our novel mouse model, combining constitutive RANK signaling with TCL1 expression in B-cells, not only promotes CLL formation but also mirrors aggressive forms of human myeloma and thereby for the first time highlights a possible role of this signaling pathway in a cell-intrinsic manner in MM development or propagation. The bone marrow niche is known to play a pro-tumorigenic role in multiple myeloma and our data highlight a new signaling node amenable to medical intervention. Our findings pave the way for further research into the mechanisms of myeloma development and treatment, with the potential to significantly improve patient outcomes.

## Material and methods

For Material and Methods please refer to Supplementary Data.

### Supplementary information

Supplementary Data

Supplementary Table 1

Supplementary Table 2

## Data Availability

For original data, please contact maike.buchner@tum.de.
