# Supplementary material for: B-cell intrinsic RANK signaling cooperates with TCL1 to induce lineage-dependent B-cell transformation
Source: Blood Cancer J. 2024 Aug 28;14(1):151. doi: 10.1038/s41408-024-01123-6 (PMC11358282; doi:10.1038/s41408-024-01123-6)
Supplement: Supplementary file 1 — Supplementary Data [file 41408_2024_1123_MOESM1_ESM.pdf]

B-cell intrinsic RANK signaling cooperates with TCL1 to induce lineage-dependent B-cell transformation

SUPPLEMENTARY DATA

MATERIAL AND METHODS

Mice. $RANK^{K240E}$ [1] were crossed to the Eμ-TCL1-transgenic mouse model [2] to generate triple-transgenic Eμ-TCL1 $RANK^{K240E}$ $CD19^{Cre}$ mice (TC-RK). For genetically deletion of CD19, we crossed our TC-RK mice to $CD19^{Cre/Cre}$ mice [3] to generate Eμ-TCL1 $RANK^{K240E}$ $CD19^{CreCre}$ ($TC\text{-}RK^{CD19KO}$), Eμ-TCL1 $CD19^{CreCre}$ (TC^{CD19KO}) and $RANK^{K240E}$ $CD19^{CreCre}$ (RK^{CD19KO}) mice. For murine MM cell transplantation, 1-2 x 10^7 splenocytes or 0.4-1.7 x 10^6 bone marrow-derived cells of diseased TC-RK mice were injected intravenously into $Rag2^{ko}$ mice purchased from Jackson laboratories. Mice were sacrificed upon sign of disease. For transplanting B2-cells, sorted $CD19^{+}CD5^{neg}$ BM-derived cells from 4-months old TC-RK mice were transplanted into $Rag2^{ko}$ mice. Engraftment and B-cell stages were monitored by analysing the GFP/CD19/CD5 cell content in the peripheral blood using flow cytometry. After 4 to 6 months, animals were sacrificed and spleens were analyzed for plasma cells. For the human MM Xenograft Model, we used NSG mice (*NOD.Cg-Prkdcscid Il2rgtm1WjI/SzJ*) purchased from Janvier laboratory as recipients for the human MM cell line L363. Animals were randomly assigned to two groups and pretreated with either anti-RANKL antibody therapy (intraperitoneal, 5 mg/kg, Bio X Cell, Lebanon, US) or PBS one day before L363 injection (intravenous, 4.5 x10^6 cells per mouse) and the day afterwards. Mice were sacrificed upon signs of paralysis or disease. Staff members at the Center of Preclinical Research (Technical University Munich, Munich) who were blinded to the experimental conditions and animal genotypes of the study assisted in determining when euthanasia was required. All animal experiments were conducted in accordance with German Federal Animal Protection Laws and approved by the Institutional Animal Care and use Committee at the Technical University of Munich.

Cell culture. The human MM cell line L363, authenticated by short tandem repeat profiling (Cell Line Authentication Service of Eurofins Genomics, Ebersberg, Germany) and murine B-cells were cultured in RPMI-Glutamax™ medium supplemented with 10 % FBS and 1% Penicillin-Streptomycin (PenStrep). Medium for naïve murine B-cells was additionally

supplemented with 1% nonessential amino acids, 1% sodium pyruvate, 1% HEPES and 0.05 mM β-mercaptoethanol. RANK-expressing BAL17 cell lines [1] were cultured in RPMI Glutamax™ medium supplemented with 10 % FBS, 0.05 mM β-mercaptoethanol and 1% PenStrep. All cells were cultured under standard conditions; at 37 °C, 5% CO_2 and 95% humidity and cell lines were regularly tested for mycoplasma by PCR.

Cell purification and stimulation. For murine *in vitro* differentiation, naïve splenic B-cells were purified using the murine B-cell isolation kit from Miltenyi Biotec. To increase the purity of untouched B-cells, Biotin conjugated antibodies against CD5 (Clone 53-7.3, BioLegend®) and CD138 (Clone 281-2, BioLegend®) were added to the Biotin-Antibody Cocktail of the kit. $2x10^5$ B-cells were seeded per 96 flat-bottom well and stimulated either with a combination of LPS (1 µg/mL, Sigma) and recombinant murine IL4 (1 ng/mL, PeproTech, Cranbury, US), LPS^{low} (100 ng/mL, Sigma) in absence or presence of recombinant murine RANKL (50 ng/mL, #462-TR-010, R&D Systems, Minneapolis, US), anti-IgM (5 µg/mL, Jackson Immuno Research) or anti-CD40 (5 µg/mL, clone FGK45, Biolegend) in technical triplicates. RANK-expressing BAL17 cell lines were stimulated for 5 minutes with either recombinant murine RANKL (50 ng/mL) and/or anti-IgM (5 µg/mL, Jackson Immuno Research) and afterwards directly harvested for Immunoblotting.

Immunoblotting. Whole cell lysates were generated by using RIPA lysis buffer supplemented with protease and phosphatase inhibitors (cOmplete™ EDTA-free protease inhibitor cocktail, Roche phosphatase inhibitor cocktail) and protein concentrations were determined by Pierce BCA Protein Assay Kit (Thermo Fisher Scientific). 15 µg of protein was used for SDS page and transferred onto nitrocellulose membrane. Membranes were blocked for 1 h in 5 % BSA in tris-buffered saline with Tween 20 (TBST, 0.1 % Tween 20) and probed with the following primary and secondary antibodies: anti-β actin (#60008-I-Ig, Proteintech), anti-AKT (#9272, Cell Signaling), anti-ERK (#9102, Cell Signaling), anti-pAKT (#4056, Cell Signaling), anti-pERK (#4370, Cell Signaling), anti-pJNK (#9251, Cell Signaling), anti-JNK (#sc-7345, Santa Cruz), anti-RANK (#sc-374360, Santa Cruz), anti-pSRC family (#2101, Cell Signaling), anti-SRC (2110, Cell Signaling), anti-pPLCγ2 (#3871, Cell Signaling), anti-PLCγ2 (#3872, Cell Signaling), anti-pIkkα/β (#2078, Cell Signaling), anti-Ikkα (#2682, Cell Signaling) and anti-α tubulin (#12351, Cell signaling), anti-rabbit IgG-HRP (#7074 Cell Signaling) and anti-mouse IgG (#7076, Cell Signaling). Blots were imaged on a ChemiDoc MP Imaging System (Bio-

Rad, Hercules, US). Densitometry quantification was acquired using the Licor Image Studio Lite software.

RBC count. Blood samples were collected in EDTA blood collection tubes and RBC count was measured using scil Vet abc Plus+ device (scil animal care company GmbH, Viernheim, Germany) according to the manufacturer's protocol.

Measurement of plasma proteins and immunoglobulins. Serum electrophoresis was conducted in the routine laboratory of the Institute of Clinical Chemistry and Pathobiology at the Klinikum rechts der Isar, Munich. Detection of immunoglobulin and proteins were performed using the LEGENDplex™ Mouse Immunoglobulin Isotyping Panel and Mouse B-cell Panel Standard manufactured by BioLegend® (San Diego, US) according to the manufacturer's instructions.

Flow cytometry. Peripheral blood and organs were harvested from mice and single cell suspensions were processed as previously described [1]. Flow Cytometry data was acquired using a FACS Canto II cytometer and analyzed with the FlowJo™ software version 10.8.1 (BD Bioscience, San Diego, US). Dead cell exclusion was obtained either by using the Zombie Aqua™ Fixable Viability Kit (BioLegend®, San Diego, US) or DAPI (1 µg/ml) (Sigma Aldrich, St. Louis, US) staining. For blocking free Fc receptors, anti-mouse CD16/32 or human Fc Receptor blocking solution (BioLegend®) were applied and cells were stained with fluorochrome-labeled antibodies according to manufacturer's information. Antibodies from BioLegend® included anti-CD19-APC-Cy7 (clone 5D3, #115530), anti-CD5-PE (clone 53-7.3, #100607), anti-Blimp1-AlexaFluor674 (clone 5E7, #150003), anti-CD138-PE (clone 281-2, #142504), anti-CD138-PerCy5.5 (clone 281-2, #142510), anti-CD45R-APC-Cy7 (RA3-6B2, #103224), anti-CD23-PE-Cy7 (B3B4, #101614), anti-CD25-PE (PC61.5, #102008), anti-CD19-APC (6D5, #115512). Anti-CD21-PE (7G6, #552957) was purchased from BD Bioscience (Franklin Lakes, US). Antibodies from eBioscience (San Diego, US) included anti-CD265-PE (clone R12-31, #12-6612-82), anti-IRF4-PerCP-efluor700 (clone 3E4, #46-9858-82), anti-IgM-PE-Cy7 (clone II/41, #25-5790-81), anti-IgD-APC (clone 11-26c (11-26), #17-5993-80), anti-CD45R-eFluor450 (RA3-6B2, #48-0452-82), anti-CD5-APC (53-7.3, #17-0051-82), anti-CD117-APC (2B8, #17-1171-81). Anti-CD265-PE (9A725, #MA1-41015) was purchased from Invitrogen (Waltham, US). Intracellular stainings were performed using the intracellular fixation & permeabilization buffer set from eBioscience.

Histology. Murine organs were fixed in 10% neutral buffered formalin for 48 hours, paraffin embedded and sectioned according to routine methods. Bones were additionally decalcified in Osteosoft® (Sigma Aldrich, St. Louis, US) for 1 week after fixation. Sections were stained with Hematoxylin-Eosin, anti-murine CD138 antibody (clone 281-2, BD Bioscience) IRF4 (M-17, Santa Cruz, Santa Cruz, US). Human MM biopsies were stained with anti-human TNFRSF11A/RANK antibody (clone 9A725, LSBio, Lynnwood, US). Slides were scanned with a Leica AT2 biosystem and both HE and IHC were analyzed and evaluated by a blinded board-certified pathologist.

RNA sequencing and differential gene expression analysis. RNA of purified plasma cells (CD138 MicroBeads, Miltenyi Biotech, Bergisch Gladbach, Germany) from RK and TC-RK mice was isolated by using a Qiagen RNeasy Mini Kit (Venlo, Netherlands) according to manufacturer's protocol. Quality control, mRNA library preparation and paired-end sequencing were subsequently performed by Novogene (Cambridge, UK) on a NovaSeq 6000 System (Illumina, San Diego, US) with a sequencing depth of 30 M reads/sample. The reference genome GRCm38.p6 was used for alignment. Differential expression analysis was performed in R (v4.2.1) with DESeq2 (v1.36) using the LRT test. A gene was considered differentially expressed if its FDR adjusted p-value was below 0.05. Gene Set enrichment analysis was performed with R (v4.2.1) using the GSEA package (v4.3.2). Analysis of gene list similarities was performed by using 'GeneOverlap' package in R (v4.3.2) [4] and Fisher's exact test was applied to determine the statistical significance.

Single-cell RNA sequencing and analysis. scRNA-seq was performed on BM flushes after RBC lysis pooled from 5-months-old TC-RK (n=4) compared to RK (n=3) mice. Pooled cell suspensions (one RK and one TC-RK) were immediately processed using the 10X Genomics (CA, USA) scRNA kit, following the manufacturer's instructions. Library preparation was performed according to the Chromium Single Cell 3′ Reagent Kit v3.1 (10X Genomics, PN-1000269) and the 3′ Feature Barcode Kit (10X Genomics, PN-1000262) according to the manufacturer's instructions. Sequencing was conducted using an Illumina NovaSeq 6000 Sequencing system at the Helmholtz Munich. Subsequently, the sequencing files were processed in accordance with the 10x Genomics workflow by the Helmholtz Core Facility.
Doublets were removed using the python package Scrublet [5]. Further downstream processing was performed using Seurat (v4.3.0) [6]. Quality control filtering was performed to eliminate low-quality cells with the following parameters: cells with fewer than 200 detected genes or ≥ 10% mitochondrial reads were removed. The remaining high-quality cells were kept for

downstream processing. Data were normalized using the *SCTransform* function, regressing out technical confounding factors (mitochondrial reads) using the *vars.to.regress* arguments. Principal component analysis (PCA) and uniform manifold approximation and projection dimensionality reduction (UMAP) was performed based on the normalized data.

Celltype annotation was performed using SingleR [7] with mouse bulk expression data from the Immunologic Genome Project (ImmGen) dataset [8] as a reference. Cell types with a low abundance (<10 cells) were eliminated, which resulted in removal of stromal cells, fibroblasts, epithelial cells, eosinophils and endothelial cells. Plasma cells were defined B-cells expressing plasma cell marker genes Sdc1 and Slamf7 as follows: Plasma <- subset (x = seurat, subset = Sdc1 > 0.5 & Slamf7 > 0.5, idents = "B-cells").

Marker genes for each of the conditions were identified using the *FindMarker* function from the Seurat package. The gene signatures employed in this study were obtained from the Molecular Signature Database (https://www.gsea-msigdb.org/gsea/index.jsp) or defined based on the literature. To characterize cell states, we computed signature scores by averaging the relative expression of gene sets. Gene-set enrichment analysis was conducted using Fgsea [9], and P values were determined by one-tailed permutation test. The Milo [10] implementation in Python (milopy package) was used to estimate changes in cell type abundance between conditions. Neighborhoods were annotated based on the predominant cell type and those neighborhoods where less than 50% of cells belonged to one cell type were labelled as "Mixed". Shown are neighborhoods with FDR<0.1.

Whole exome sequencing and analysis. DNA of MACS purified splenocytes using the CD138 plasma cell kit (Miltenyi Biotech) from diseased TC-RK mice (n=7) was isolated by using the DNeasy® Blood & Tissue Kit (Qiagen). Quality control, mRNA library preparation and sequencing were carried out by Novogene. The sequencing was conducted on a NovaSeq System, utilizing a paired-end 150bp read format with a S4 flow cell.

Analysis was conducted following GATK best practice suggestions based on the established analysis pipeline MoCaSeq [11]. In brief, Trimmomatic (v0.39) was used to trim raw sequencing reads before mapping. Reads were aligned using BWA-MEM 0.7.17 with default settings and the reference genome GRCm38.p6. PCR duplicates were identified with samblaster (v0.1.26) and sambamba (v0.7.0), together with Picard tools (v2.20.0) and realignment around indels was performed using the GATK toolkit (v4.2.0.0). Somatic mutations were called by using Mutect2 with default settings, based on the paired case and control sample. Potential somatic events were filtered for SNPs by excluding single nucleotide variants (SNV) which

were reported in the Mouse Genome Project SNP database (v5). Furthermore, 'LearnReadOrientationModel' was used for exclusion of SNVs marked as strand or PCR bias artefact and annotation of somatic events was performed with SNPeff (version 4.3). SNVs with a low predicted impact and variants at non-exonic sites were excluded from further analysis. Data was deposited to ENA PRJEB73315. A comparative analysis investigated MM mutation profiles between the mouse model and the human myeloma dataset. The mutation profile of 205 samples was employed from previously conducted study in 2014 [12]. This dataset was sourced from cBioPortal.

Copyratios were defined using CNVKit (v0.9.9). Calling was conducted using the "batch" command of the CNVKit pipeline including read coverage estimation in target and antitarget regions, normalization and segmentation. The probe regions of the whole-exome sequencing kit served as on-target regions.

Statistical Analysis. Statistical analyses were performed using GraphPad Prism Version 8.0, GraphPad Software Inc. Statistical significance between two groups was analyzed with paired or unpaired two-tailed Student's t test, while ordinary one-way ANOVA with Turkey's multiple comparison test was used for more than two groups. Kaplan-Meier plot was used for probability of survival and Log-Rank (Mantel Cox) testing was used for comparison of cohorts. Moreover, statistical methods were not used to determine the sample size in advance. All data are presented as mean ± standard deviation. $P<0.05$ was considered as statistically significant.

SUPPLEMENTARY REFERENCES

1. Alankus, B., V. Ecker, N. Vahl, M. Braun, W. Weichert, S. Macher-Goppinger, T. Gehring, et al., *Pathological RANK signaling in B cells drives autoimmunity and chronic lymphocytic leukemia.* J Exp Med, 2021. **218**(2).
2. Bichi, R., S.A. Shinton, E.S. Martin, A. Koval, G.A. Calin, R. Cesari, G. Russo, et al., *Human chronic lymphocytic leukemia modeled in mouse by targeted TCL1 expression.* Proc Natl Acad Sci U S A, 2002. **99**(10): p. 6955-60.
3. Rickert, R.C., J. Roes, and K. Rajewsky, *B lymphocyte-specific, Cre-mediated mutagenesis in mice.* Nucleic Acids Res, 1997. **25**(6): p. 1317-8.
4. Shen, L.J.R.P., *GeneOverlap: an R package to test and visualize gene overlaps.* 2014.
5. Wolock, S.L., R. Lopez, and A.M. Klein, *Scrublet: Computational Identification of Cell Doublets in Single-Cell Transcriptomic Data.* Cell Syst, 2019. **8**(4): p. 281-291 e9.
6. Hao, Y., S. Hao, E. Andersen-Nissen, W.M. Mauck, 3rd, S. Zheng, A. Butler, M.J. Lee, et al., *Integrated analysis of multimodal single-cell data.* Cell, 2021. **184**(13): p. 3573-3587 e29.

7. Aran, D., A.P. Looney, L. Liu, E. Wu, V. Fong, A. Hsu, S. Chak, et al., *Reference-based analysis of lung single-cell sequencing reveals a transitional profibrotic macrophage.* Nat Immunol, 2019. **20**(2): p. 163-172.
8. Heng, T.S., M.W. Painter, and C. Immunological Genome Project, *The Immunological Genome Project: networks of gene expression in immune cells.* Nat Immunol, 2008. **9**(10): p. 1091-4.
9. Korotkevich G, S.V., Sergushichev A (2019). "Fast gene set enrichment analysis." bioRxiv. doi:10.1101/060012, http://biorxiv.org/content/early/2016/06/20/060012.
10. Dann, E., N.C. Henderson, S.A. Teichmann, M.D. Morgan, and J.C. Marioni, *Differential abundance testing on single-cell data using k-nearest neighbor graphs.* Nat Biotechnol, 2022. **40**(2): p. 245-253.
11. Lange, S., T. Engleitner, S. Mueller, R. Maresch, M. Zwiebel, L. Gonzalez-Silva, G. Schneider, et al., *Analysis pipelines for cancer genome sequencing in mice.* Nat Protoc, 2020. **15**(2): p. 266-315.
12. Lohr, J.G., P. Stojanov, S.L. Carter, P. Cruz-Gordillo, M.S. Lawrence, D. Auclair, C. Sougnez, et al., *Widespread genetic heterogeneity in multiple myeloma: implications for targeted therapy.* Cancer Cell, 2014. **25**(1): p. 91-101.
13. Larrayoz, M., M.J. Garcia-Barchino, J. Celay, A. Etxebeste, M. Jimenez, C. Perez, R. Ordonez, et al., *Preclinical models for prediction of immunotherapy outcomes and immune evasion mechanisms in genetically heterogeneous multiple myeloma.* Nat Med, 2023. **29**(3): p. 632-645.

SUPPLEMENTARY FIGURE LEGENDS

Supplementary Figure 1: Characterization of TC-RK mice and their myeloma cells

a) Dot plot graph depicts spleen (SP) size in centimeters (cm) of diseased or aged mice with indicated genotypes (n=3-9 per genotype). **b)** Dot plot graph depicts liver (LI) weight in gram (g) of diseased or aged mice with indicated genotypes (n=3-8). **c)** Geometric mean (geo. mean) of forward scatter area (FSC-A), surface RANK, CD138, B220, IgM and IgD, as well as intracellular IRF4 and BLIMP1 from $GFP^{hi}CD19^{neg}$ cells (green) compared to $GFP^{+}CD19^{+}$ cells (black) from TC-RK mice (n=4-5). **d)** Representative images of H&E and immunohistochemistry (CD138, IRF4) of spleens of diseased TC-RK and control mice (scale bars: overview=1mm, detailed images: 40x magnification). **e)** Kaplan-Meier OS analysis of $Rag2^{ko}$ mice (n=3) transplanted with TC-RK bone marrow cells derived from two different sick donor mice compared to $Rag2^{ko}$ control mice. **f)** Percentages of $GFP^{hi}CD19^{neg}$ cells and $GFP^{+}CD19^{+}$ cells of viable cells from bone marrow (BM), spleen (SP), peripheral blood (PB) and liver (LI) isolated from diseased $Rag2^{ko}$ mice (n=3) transplanted with TC-RK bone marrow cells derived from two different donor mice. Statistical analysis was performed using the one-way ANOVA with Tukey correction for multiple comparison and Student's t-test. P values are indicated in respective graphs. All data are presented as mean ± standard deviation.

<u>Supplementary Figure 2: *In vivo* and *in vitro* differentiation capacity of B-cells from TC-RK mice</u>

a) Bone marrow B-cell developmental stages of six-weeks-old TC-RK (n=5) and control mice (n=5-6 per genotype) were assessed by flow cytometry as follows: B220^{+}CD19^{+} (of living cells), IgM^{neg} (B220^{+}CD19^{+}), pre B (CD25^{+} cKit^{neg}, IgM^{neg}, B220^{+}CD19^{+}), pro B-cells (cKit^{+}CD25^{neg}, IgM^{neg}, B220^{+}CD19^{+}), immature (IgM^{+}B220^{+}CD19^{+}) recirculating (IgM^{+}B220^{high}, CD19^{+}). Pooled data from three individual experiments. **b)** Percentage of splenic CD19^{+}CD5^{+} of viable cells pre-gated on CD19^{+}CD5^{neg} (right) from 6-weeks-old TC-RK (n=6) and control mice (n=4-6 per genotype). Pooled data from three individual experiments. **c**) Percentages of splenic CD21^{+}CD23^{neg} marginal zone (MZ) B-cells pre-gated on CD19^{+}CD5^{neg} (right) from six-weeks-old TC-RK (n=6) and control mice (n=4-6). Pooled data from three individual experiments. **d)** Quantification of IgA immunoglobulin isotype in supernatants from in vitro differentiated naïve B-cells, derived from animals with indicated genotype (n=3-5) and stimulated for four days with LPS^{low}, by flow cytometry-based multiplex immunoassay. Pooled data from two different experiments. **e)** Experimental set up of *in vitro* differentiation of MACS-isolated naïve B-cells using a combination of LPS (1µg/mL) and 1 ng/mL recombinant murine IL4 or anti-IgM (5µg/mL) or anti-CD40 (5µg/mL). B-cell stages were analyzed by flow cytometry four days after stimulation. Graphic was created BioRender.com. **f)** Percentages of CD138^{+}B220^{low} plasma cells of living cells after *in vitro* differentiation with LPS and IL4 determined by flow cytometry four days after stimulation. Biological replicates were pooled from two individual experiments. **g)** Percentages of CD138^{+}B220^{low} plasma cells of living cells after *in vitro* stimulation with anti-IgM determined by flow cytometry after four days. Biological replicates were pooled from two individual experiments. **h)** Percentages of CD138^{+}B220^{low} plasma cells of living cells after four days *in vitro* stimulation with anti-CD40 determined by flow cytometry. Biological replicates were pooled from two individual experiments. Statistical analysis was performed using the one-way ANOVA with Tukey correction for multiple comparison. P values and not significant (ns) results are indicated in respective graphs. All data are presented as mean ± standard deviation.

<u>Supplementary Figure 3: Signaling transduction after RANK and/or BCR stimulation in RANK-overexpressing BAL17 B-cell lymphoma cells</u>

a) BAL17 pMIG, BAL17 RANK^{wt} and BAL17 RANK^{K240E} cells were stimulated with either murine RANKL (50 ng/mL) and/or aIgM (5 µg/mL) for 5 minutes. Representative immunoblots

blotted for the indicated phospho- and total proteins and a loading control from one out of three independent experiments. **b)** Densitometry quantification of phosphoproteins to their total counterparts shown in a) and normalized to the unstimulated control (-RANKL/-aIgM) from three independent experiments. Student's t-test was applied on indicated conditions to highlight effects of RANKL stimulation in $RANK^{wt}$- and $RANK^{K240E}$-overexpressing BAL17 cell lines.

Supplementary Figure 4: T and B-cell immunophenotyping within the bone marrow niche of TC-RK mice

a) UMAP plots of unsupervised clustering analysis of B-cells and plasma cells (defined by *Sdc1* and *Slamf7* expression). Cells of individual UMAP plots are color coded according to *Ptprc, Pax5*, *Ebf1*, *Sqstm1*, *CD74*, and *Mzb1* gene expression respectively. **b)** TC-RK cells within the B and plasma cell subcluster show a higher plasma cell score. **c)** TC-RK cells with in the T cell subcluster show a higher CD8 exhaustion (Tex, left) and activation phenotype (right) as compared to RK-derived bone marrow cells.

Supplementary Figure 5: Overlap between TC-RK-induced myeloma and other murine-induced and human Multiple Myeloma

a) Table depicts differential gene expression similarities analysis including p values and odds ratios between myeloma cells from TC-RK mice compared to myeloma cells from various multiple myeloma mouse models [13]. Fisher's exact test was applied and both p values and odd ratios are depicted. **b)** Table of histological evaluation by a certified pathologist of RANK expression in human MM patients according to their percentage of myeloma cell infiltration, percentage of $RANK^{+}$ stained myeloma cells and staining intensity.

Suppl.Figure 1

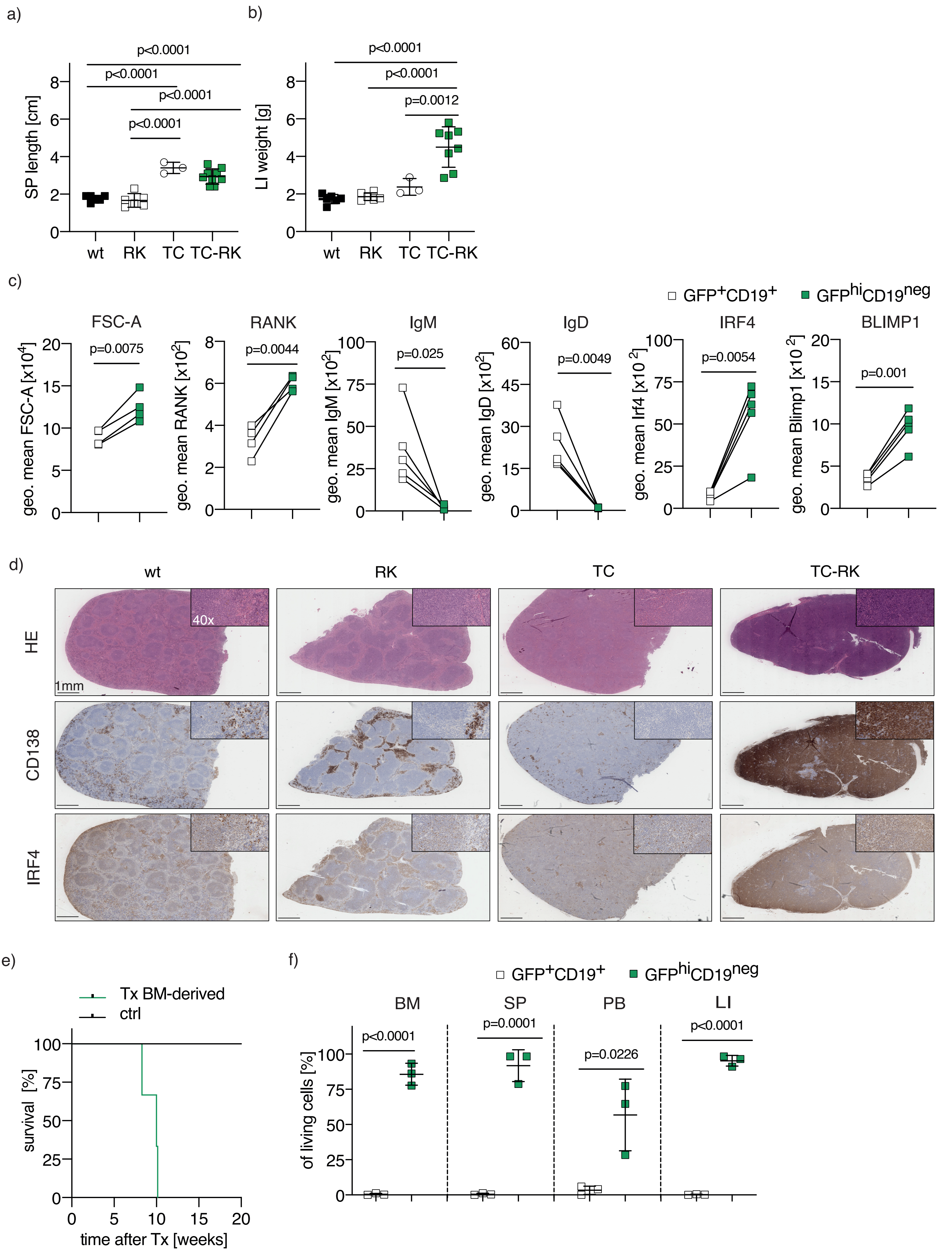

Suppl.Figure 2

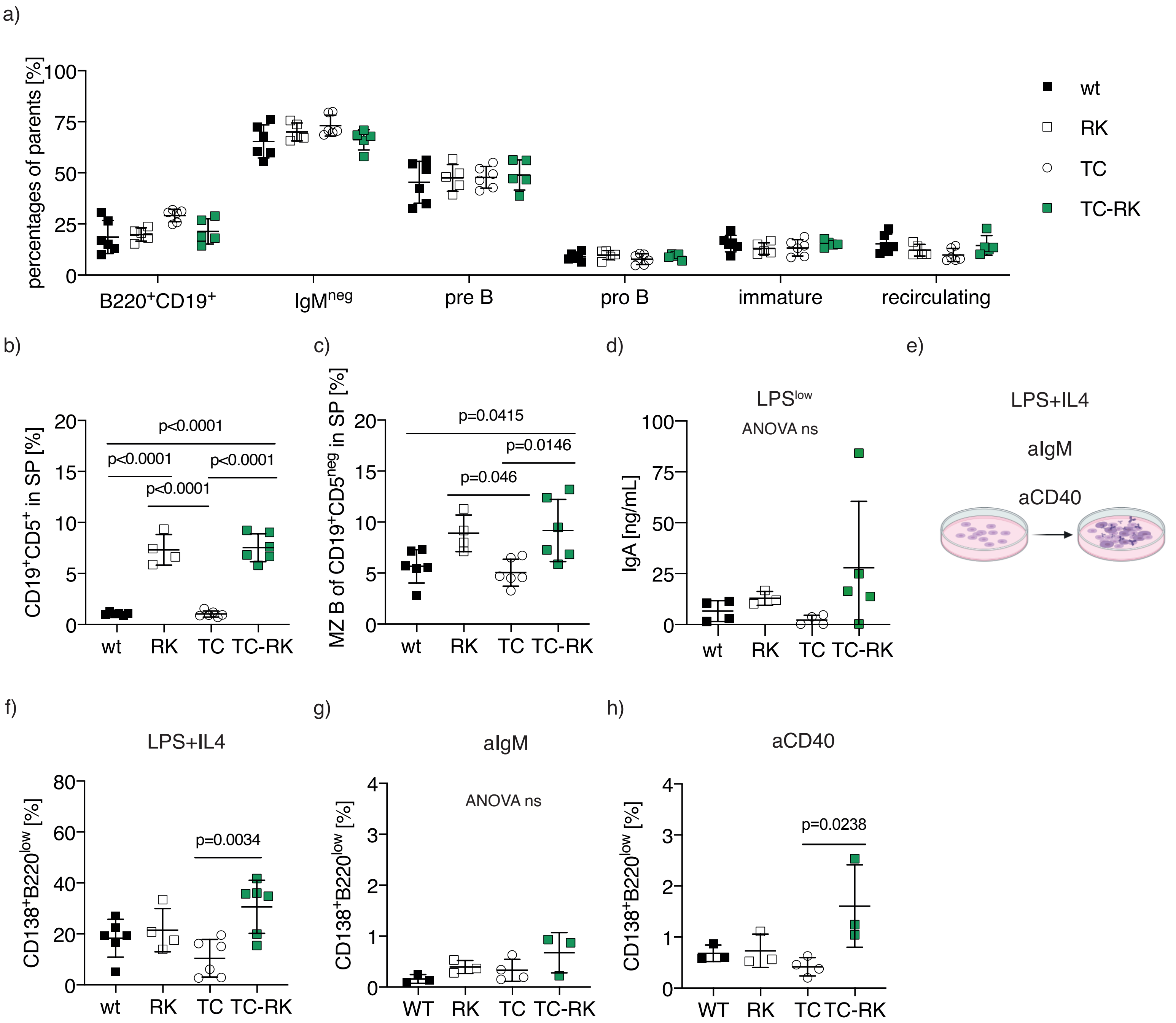

Suppl.Figure 3

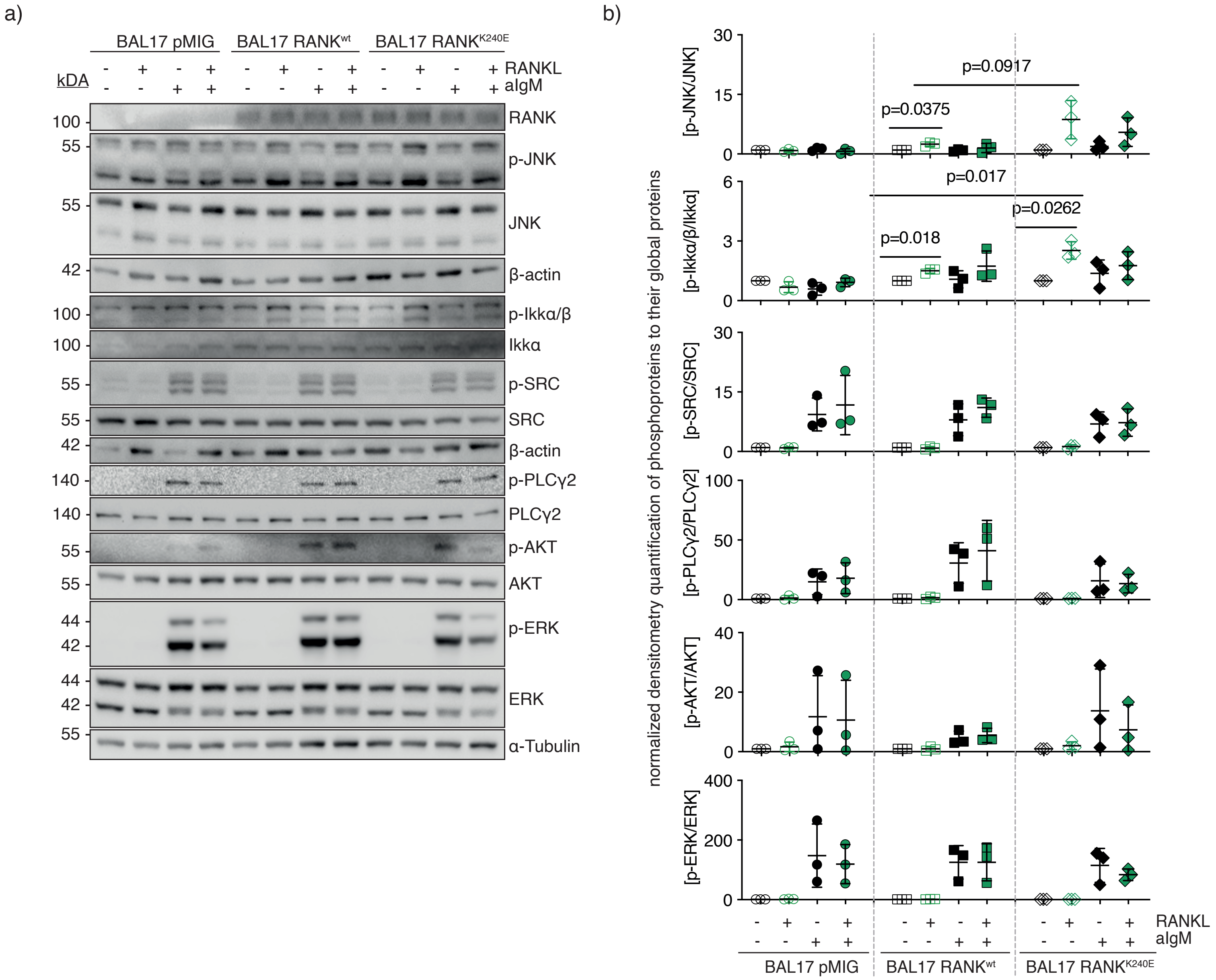

Suppl.Figure 4

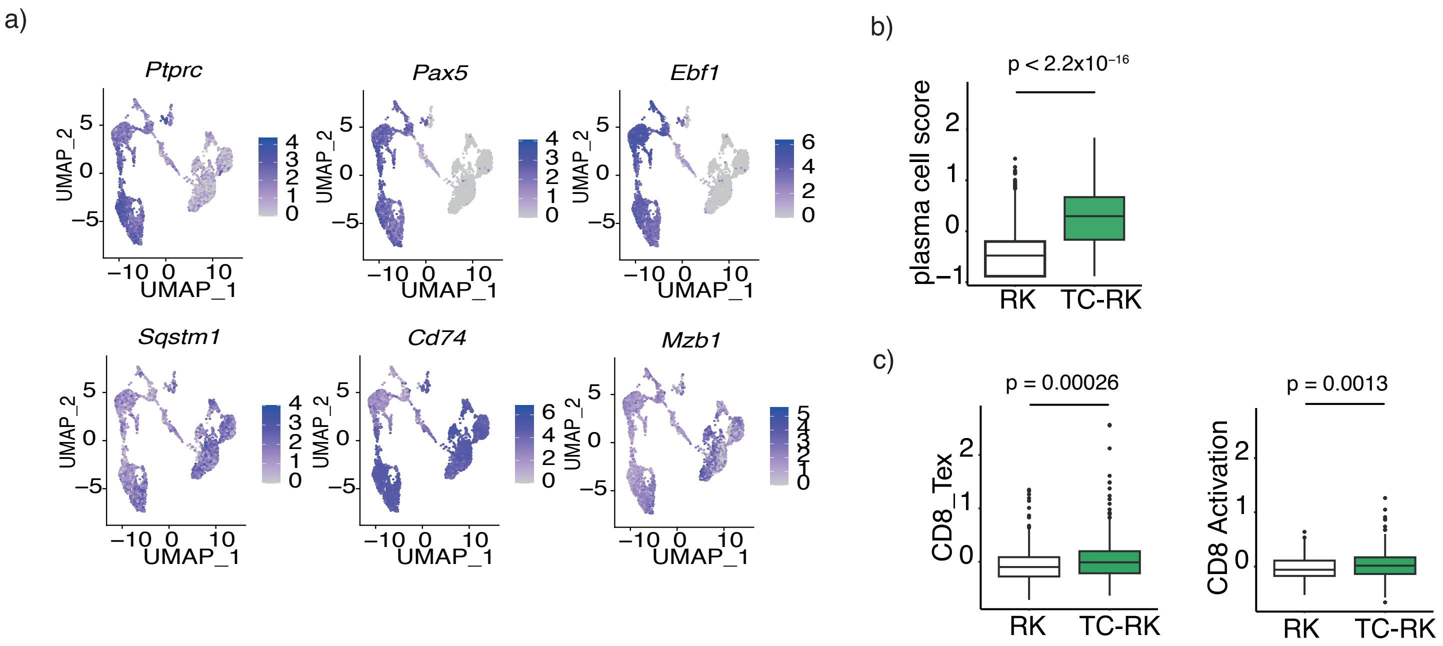

Suppl.Figure 5

a)

mouse model	p value	odds ratio
$MI_{c\gamma 1}$	$1.9x10^{-116}$	2.3
$Maf\text{-}MI_{c\gamma 1}$	$8.8x10^{-93}$	2.3
$BI_{c\gamma 1}$	$2.9x10^{-129}$	3.0
$Kras\text{-}BI_{c\gamma 1}$	$1.8x10^{-246}$	3.7
$Trp53\text{-}BI_{c\gamma 1}$	$9.1x10^{-182}$	2.6
$CyclinD1\text{-}BI_{c\gamma 1}$	$1.2x10^{-98}$	2.1
$Mmset\text{-}BI_{c\gamma 1}$	$1x10^{-158}$	2.6
$Maf\text{-}BI_{c\gamma 1}$	$1.4x10^{-153}$	3.0

b)

MM patient ID	infiltration [%]	$RANK^+$ myeloma cells	intensity
11/18127	40-50	>90	moderate
15/15411	40-50	10	weak
16/1995	50-60	>90	weak
16/11789	70	>90	moderate
20/8767	70	>90	moderate
16/19408	95	30	weak
16/18518	60-70	3	weak
20/13015	95	40	weak
21/2084	40	60	weak
21/15274	40	30	weak
